# Metastatic Mucinous Adenocarcinoma of the Prostate with PSA Value of 8.6 ng/mL at 5-Year-Followup after Prostatectomy, Radiotherapy, and Androgen Deprivation

**DOI:** 10.1155/2014/218628

**Published:** 2014-01-29

**Authors:** Christos Kalaitzis, Michael Koukourakis, Stilianos Giannakopoulos, Alexandra Giatromanolaki, Efthimios Sivridis, Athanasios Bantis, Stavros Touloupidis

**Affiliations:** ^1^Department of Urology, University of Thrace, 68100 Alexandroupolis, Greece; ^2^Department of Radiotherapy, University of Thrace, 68100 Alexandroupolis, Greece; ^3^Department of Pathology, University of Thrace, 68100 Alexandroupolis, Greece

## Abstract

*Introduction*. Mucinous adenocarcinoma of the prostate is a rare variant of prostate cancer. Its malignant potential and the clinical course of the affected patients remain, by and large, controversial. No data exist about the course of metastatic mucinous adenocarcinoma of the prostate. *Case Presentation*. This case report describes the excellent clinical course of a 68-year-old patient with metastatic mucinous adenocarcinoma of the prostate, treated by radical prostatectomy, irradiation, and androgen deprivation. *Conclusion*. In our case, mucinous adenocarcinoma of the prostate does not appear to behave differently than acinar prostate cancer. Its malignant potential is dependent on its Gleason score.

## 1. Introduction

Mucinous adenocarcinoma of the prostate is one of the least common morphological variants of prostate cancer, defined by the presence of pools of extraluminal mucin involving at least 25% of the tumor volume at prostatectomy. Mucinous adenocarcinoma is very rare with an incidence of approximately 0.2% [1–3].

Grading and prognosis of this rare variant of prostate cancer remain controversial [2, 4, 5].

In previous publications, urologic pathologists have proposed that all colloid carcinomas should be considered, by definition, as a Gleason 8 [1, 4, 5].

Recent articles, however, suggest that the Gleason score should be assigned on the basis of the underlying glandular architecture, irrespective of the presence of mucin [6, 7].

Several case reports have described an aggressive clinical course of mucinous adenocarcinoma of the prostate [1, 8–11].

Yet, Osunkoya et al. in a series of 47 cases showed an excellent prognosis for mucinous adenocarcinoma of the prostate [12].

There are no data about the course of metastatic mucinous adenocarcinomas of the prostate.

We report herewith the clinical course of a mucinous adenocarcinoma of the prostate with metastasis to iliac lymph nodes at a PSA value of 8.6 ng/mL treated by radical prostatectomy, followed by irradiation and androgen deprivation according to Vancouver protocol.

## 2. Case

**Figure 1:**
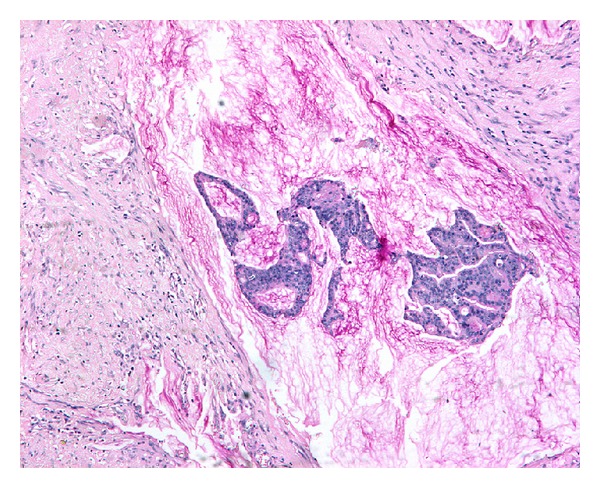
Mucinous adenocarcinoma of the prostate (alcian blue/PAS).

A 68-year-old Caucasian man was admitted to hospital with a diagnosis of prostate cancer (Gleason score 4 + 3 = 7, PSA value 8.6 ng/mL), after taking a biopsy in an external urological surgery. CT scan and bone scan were negative for metastases. We treated the patient with retropubic open prostatectomy and standard iliac lymphadenectomy. Frozen section of lymph nodes was not performed because of a PSA < 10 ng/mL. Histopathological examination confirmed the presence of a prostatic adenocarcinoma with extraluminal mucinous pools involving >80% of the tumor volume. The final Gleason score given was 5 + 4 = 9 and the stage pT3b (Figure 1). The lymph nodes of the left iliac fossa were involved.

The first measurement of PSA 4 weeks after surgery showed a value of 9.2 ng/mL. A new CT abdominal scan revealed a mass of 5 cm diameter in the field of the left iliac junction, while the bone scan remained free of any metastatic involvement. We started androgen deprivation therapy with concomitant boost accelerated hyperfractionated radiotherapy of the iliac lymph nodes with a dose of 2.7 Gy × 14 and the lymph mass with an additional 0.7 Gy × 15.

Six months after the end of the radiotherapy, the PSA value was 0.001 ng/mL. We stopped androgen deprivation therapy and measured PSA and testosterone value every 3 months. The last measurement, 5 years after radical prostatectomy, revealed a PSA value of 0,06 ng/mL and a total testosterone of 2,05 ng/mL.

It is our plan to restart androgen deprivation therapy at PSA value of ≥2 ng/mL.

## 3. Discussion

Intraluminal mucin is seen in almost a third of prostate adenocarcinomas [13]. However, a diagnosis of mucinous adenocarcinoma is made only when extraluminal pools of mucin involve at least 25% of the tumor volume at prostatectomy. Otherwise, it is better to speak of a “prostate adenocarcinoma with mucinous features” [6]. The biological behaviour of a mucinous adenocarcinoma of the prostate with regard to PSA production and metastasis is similar to that of a usual acinar adenocarcinoma. However, several case reports have shown that patients with mucinous adenocarcinoma of the prostate may have a variable outcome in terms of hormone responsiveness and recurrence [2, 3, 8–10]. Furthermore, its prognosis relative to a conventional carcinoma remains controversial for many authors found that mucinous adenocarcinoma of the prostate is more aggressive than nonmucinous prostate cancer [2, 8–11], but others found that the reverse is valid [3, 5, 14].

Along these lines, Osunkoya et al. demonstrated an excellent prognosis for mucinous adenocarcinoma in a series of 47 cases [12]. These investigators ignored the extracellular mucin and graded the tumor on the basis of the underlying architectural pattern. They found that in 15% of the prostate the entire tumor was considered as Gleason 6 and the rest as Gleason 7. Saito and Iwaki reviewed 87 previously reported cases of mucin-producing carcinomas of the prostate and found that the prognosis of this variant depended on the presence of signet-ring cells [11]. Mucinous carcinomas with signet-ring cells had a very poor prognosis in contrast to signet-ring cell carcinomas. In another study, Lane et al. compared 14 patients with mucinous adenocarcinoma of the prostate with 18 other patients who had prostate cancer with focal mucinous features and reported that the former behaved clinically in a very similar fashion to the latter [5]. Johnson et al. showed that ERG gene expression in mucinous prostate cancer and prostatic adenocarcinoma with mucinous features is similar to rates of expression in acinar prostate adenocarcinomas [6]. It is probable that the high metastatic potential of prostate cancer in our patient was a result of the high Gleason score and not of the presence of mucin pools in more than 80% of the tumor. His excellent clinical course after radical prostatectomy, radiotherapy, and androgen deprivation, despite lymph nodes metastasis, is consistent with the observation of other authors who reported survival benefit of radical prostatectomy in lymph node positive prostate cancer patients [15].

## 4. Conclusion

The clinical course of a mucinous adenocarcinoma of the prostate seems not to be different than that of an acinar prostate adenocarcinoma and is presumably dependent on the Gleason score and not on the presence of mucin pools at prostatectomy.
